# Immunoprecipitation of Reporter Nascent Chains from Active Ribosomes to Study Translation Efficiency

**DOI:** 10.21769/BioProtoc.4821

**Published:** 2023-09-20

**Authors:** Roberta Cacioppo, Catherine Lindon

**Affiliations:** Department of Pharmacology, University of Cambridge, Cambridge, UK

**Keywords:** Reporter assay, UTR regulation, mRNA translation, Transient transfection, Nascent chain immunoprecipitation, One-step RT-qPCR

## Abstract

The study of translation is important to the understanding of gene expression. While genome-wide measurements of translation efficiency (TE) rely upon ribosome profiling, classical approaches to address translation of individual genes of interest rely on biochemical methods, such as polysome fractionation and immunoprecipitation (IP) of ribosomal components, or on reporter constructs, such as luciferase reporters. Methods to investigate translation have been developed that, however, require considerable research effort, including addition of numerous features to mRNA regions, genomic integration of reporters, and complex data analysis. Here, we describe a simple biochemical reporter assay to study TE of mRNAs expressed from a transiently transfected plasmid, which we term Nascent Chain Immunoprecipitation (NC IP). The assay is based on a plasmid expressing an N-terminally Flag-tagged protein and relies on the IP of Flag-tagged nascent chains from elongating ribosomes, followed by quantitative reverse transcription polymerase chain reaction (RT-qPCR) quantification of eluted mRNA. We report that elution of mRNA following IP can be achieved by treatment with puromycin, which releases ribosome-mRNA complexes, or with purified Flag peptide, which instead releases nascent chain-ribosome-mRNA complexes. In the example described in this protocol, untranslated regions (UTRs) of a gene of interest were used to flank a FlagVenus coding sequence, with the method allowing to infer UTR-dependent regulation of TE. Importantly, our method enables discrimination of translating from non-translating mRNAs. Additionally, it requires simple procedures and standard laboratory equipment. Our method can be used to test the effect of regulators, such as microRNAs or therapeutic drugs or of various genetic backgrounds, on translation of any user-selected mRNA.

Key features

• The novel NC IP protocol builds upon a previously published method for detection of mRNA-binding proteins (Williams et al., 2022).

• The NC IP protocol is adapted for detecting mRNA actively undergoing translation.

• The method uses mammalian cell culture but could be adapted to multiple organisms, including budding yeast (*S. cerevisiae*).

**Graphical overview**


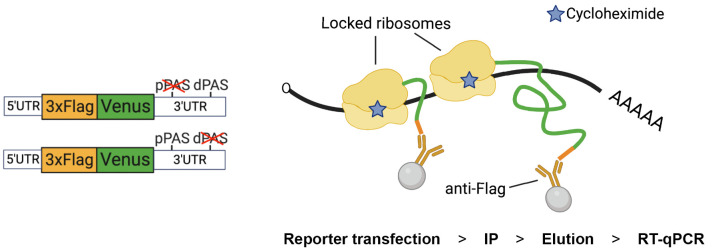


**Design of the Nascent Chain Immunoprecipitation (NC IP) reporter and assay.** *Left*. The construct carries a 3× Flag tag at the N-terminal end of Venus protein (FlagVenus). In this example, the reporter is adapted to study untranslated regions (UTR)-dependent expression by flanking FlagVenus coding sequence with UTRs of Aurora kinase A (AURKA) mRNA (depicted reporters refer to Cacioppo et al., 2023, Figure 3). The depicted reporters carry mutations in the proximal (p) or distal (d) polyadenylation signal (PAS). *Right*. Following reporter transfection, ribosomes are locked onto reporter mRNA by treating cells with cycloheximide (CHX), which prevents ribosome run-off and additional rounds of elongation, before cell lysis and immunoprecipitation (IP) of FlagVenus nascent chains via anti-Flag beads. Reporter mRNAs are then eluted, isolated, and quantified by RT-qPCR.

## Background

Translation is the process of protein synthesis using messenger RNAs (mRNAs) as template molecules, occurring in ordered steps (Blanchet and Ranjan, 2022), typically modulated by 5′ and 3′ untranslated regions (UTRs) (Hinnebusch et al., 2016; Mayr, 2019). The gold standard method for measurements of translation efficiency (TE) is ribosome profiling (Ingolia et al., 2009). However, this method allows for genome-wide analysis only, whereas, in some cases, specific investigation of TE of individual mRNAs might be of interest. A typical approach to understanding TE of specific mRNAs is to complement measurements of protein abundance with measurements of mRNA stability. Luciferase reporters are often used, with the luciferase coding sequence flanked by UTR(s) of interest. This approach, however, ignores influences of protein maturation and degradation rates. Alternatively, a measure of TE is provided by quantifying target mRNAs recovered after fractionation of polysomes or immunoprecipitation (IP) of ribosomal components. However, although highly translating mRNAs are loaded with a high number of ribosomes (Guttman et al., 2013; Ingolia et al., 2014; Liang et al., 2018), it is highly debatable whether a high number of ribosomes on transcripts is per se a robust indicator of high TE specifically of coding regions. Untranslated regions may instead be loaded with ribosomes actively engaged in translation (Ingolia et al., 2014); alternatively, transcripts may be bound by ribosomes while lacking coding capacity (Guttman et al., 2013). Additionally, neither approach provides information regarding which part of the endogenous mRNA is undergoing translation. This is relevant given that a multiplicity of mRNAs contain upstream open reading frames (Renz et al., 2020). Advanced imaging- and single cell–based methods have been developed (Biswas et al., 2019). Genetically encoded reporters enable visualization of translation but require co-transfection of multiple factors and intricate cloning for the addition of extensive features to reporter UTRs, e.g., arrays of stem–loops. Also, the requirement for sophisticated imaging platforms and complex data analysis are some other limitations of these recent methods.

Here, we describe a simple yet effective alternative method that we recently introduced to assess TE of mRNAs of interest as dictated by UTRs (Cacioppo et al., 2023). Our assay is based on IP of Flag-tagged nascent chains, ensuring exclusion of non-translating mRNAs, which has already been proved an efficient approach (Raue et al., 2007; Aviner et al., 2013; Zhang et al., 2013). We have found the method particularly useful to measure the role of 3′UTRs, and it could be expanded to probe the role of regulators such as miRNAs, or of various genetic backgrounds, on translation. In addition, since our assay measures TE of transfected reporters, the translation of any specific segments of a given mRNA can be assessed independently. However, although our assay requires simple protocol steps, standard laboratory equipment, and straightforward data analysis, it is more laborious than luciferase assays and not suitable for handling a large number of samples. In addition, contrarily to the polysome fractionation and ribosomal components IP methods, our assay cannot assess TE of multiple mRNAs simultaneously. Nonetheless, we believe that our assay is a valuable addition to the tools available to researchers for the study of translation of mRNAs of interest.

## Materials and reagents

**Biological materials**

1. Human osteosarcoma epithelial cell line (U2OS) or other cell lines
2. Plasmids expressing 3× Flag-tagged reporter protein flanked by UTRs of interest from Cacioppo et al. (2023).
3. RT-qPCR forward (CTGACCCTGAAGCTGATCT) and reverse (GCATGGCGGACTTGAAGAAG) primers targeting the Venus coding sequence on the reporter mRNA

**Reagents**

1. Cell culture medium [DMEM supplemented with 10% fetal bovine serum (FBS), 200 μM GlutaMAX-1, 100 U/mL penicillin, 100 μg/mL streptomycin, and 250 ng/mL fungizone] or other media as appropriate for the selected cell line
2. Phosphate-buffered saline (PBS) (Severn Biotech, catalog number: 207410)
3. Trypsin/EDTA solution (VWR, catalog number: LZBE02-007E)
4. Neon transfection system 100 μL kit (Thermo Fisher Scientific, catalog number: MPK10025)
5. Hanks’ balanced salt solution (HBSS) (Hyclone, catalog number: 10739674)
6. RNaseZap (Sigma, catalog number: R2020)
7. UltraPure DNase/RNase-free distilled water (Thermo Fisher Scientific, catalog number: 10977035)
8. Protector RNase inhibitor (Roche, catalog number: 3335399001)
9. EDTA (Invitrogen, catalog number: 10458654)
10. Complete Mini EDTA-free protease inhibitor (Roche, catalog number: 11836170001)
11. Tris-HCl pH 7.5 (Fisher Scientific, catalog number: BP1757-100)
12. Lithium chloride (LiCl) (Sigma, catalog number: L7026)
13. Tris-buffered saline (TBS) (Sigma, catalog number: T5912)
14. Cycloheximide (CHX) (Sigma, catalog number: 01810)
15. Triton X-100 (VWR, catalog number: 28817.295)
16. Anti-Flag M2 magnetic beads (Sigma, catalog number: M8823)
17. Puromycin (Alfa Aesar, catalog number: J67236.XF)
18. 3× Flag peptide (Sigma, catalog number: F4799)
19. Ethanol (absolute) (Sigma, catalog number: 24105-2.5L-M)
20. Luna Universal One-Step RT-qPCR kit (NEB, catalog number: E3005S)
21. Monarch RNA Cleanup kit (NEB, catalog number: T2040L)

**Solutions**

1. 3× Flag peptide elution buffer (manufacturer’s instructions)
2. Lysis buffer (see Recipes)
3. Wash buffer (see Recipes)
4. Puromycin elution buffer (see Recipes)

**Recipes**

**Critical:** Prepare all solutions in RNase-free conditions.

Note: All solutions should be prepared fresh. However, buffers can be stored at 4 °C, provided that some reagents are added just before the relative experimental step (denoted with “*”).

1. **Lysis buffer**
  **Table:** 

Reagent	Final concentration	Quantity
Tris-HCl (1 M, pH 7.5)	100 mM	250 μL
LiCl (8 M)	500 mM	156 μL
EDTA (0.5 M)	10 mM	50 μL
CHX (20 mg/mL)*	0.1 mg/mL	12.5 μL
Triton X-100 (10%)	0.1%	25 μL
Protease inhibitor*	1×	Dissolve ¼ tablet directly in lysis buffer
Protector RNase inhibitor (40 U/μL)*	0.1 U/μL	6.25 μL
H_2_O	n/a	2 mL
Total	n/a	2.5 mL
2. **Wash buffer**
  **Table:** 

Reagent	Final concentration	Quantity
Tris-HCl (1 M, pH 7.5)	10 mM	30 μL
LiCl (8 M)	600 mM	225 μL
EDTA (0.5 M)	1 mM	6 μL
CHX (20 mg/mL)*	0.1 mg/mL	15 μL
Protector RNase inhibitor (40 U/μL)*	0.1 U/μL	7.5 μL*
H_2_O	n/a	2,717 μL
Total	n/a	3 mL
3. **Puromycin elution buffer**
  **Table:** 

Reagent	Final concentration	Quantity
Tris-HCl (1 M, pH 7.5)	10 mM	6 μL
LiCl (8 M)	600 mM	45 μL
EDTA (0.5 M)	1 mM	1.2 μL
Protector RNase inhibitor (40 U/μL)*	0.1 U/μL	1.5 μL*
Puromycin (200 μM)	20 mM	6 μL
H_2_O	n/a	540 μL
Total	n/a	600 μL

**Laboratory supplies**

1. Ice
2. Tissue culture dishes (15 cm) (Nunc, catalog number: 168381)
3. Tissue culture dishes (10 cm) (Nunc, catalog number: 150350)
4. Sterile pipette filter tips (10, 200, 1,000 μL) (Starlab, catalog number: S1122)
5. 10 mL plastic pipettes (Fisher Scientific, catalog number: 11839660)
6. 50 mL centrifuge tubes (Greiner, catalog number: 227285)
7. Sterile and RNase-free 1.5 mL centrifuge tubes
8. Cell scrapers (Fisher, catalog number: 11597692)
9. 96-well reaction plates (Thermo Fisher Scientific, catalog number: 4346906)
10. Optical adhesive films (Thermo Fisher Scientific, catalog number: 4360954)

## Equipment

1. Pipettes (10 μL, 200 μL, 1,000 μL) (Gilson)
2. Pipetboy (Integra)
3. Centrifuge (Thermo Fisher Scientific, catalog number: 15868722)
4. MiniSpin centrifuge (Eppendorf, catalog number: 5452000060)
5. Neon Transfection System (Thermo Fisher Scientific, catalog number: MPK5000)
6. Neon Transfection System Pipette (Thermo Fisher Scientific, catalog number: MPP100)
7. Micro-volume spectrophotometer NanoDrop^TM^ Lite (Thermo Fisher Scientific)
8. Tube rotator (Fisherbrand, catalog number: 88861050)
9. Magnetic rack (Invitrogen)
10. StepOnePlus Real-Time PCR System (Applied Biosystems, catalog number: 4376600)
11. Ice buckets
12. 4 °C fridge and -20 °C freezer

## Software and datasets

Microsoft Excel (Microsoft Corporation) for analysis of RT-qPCR data and creation of graphs. Can be substituted by R or any other software for data analysis.

## Procedure

**Figure 1.**
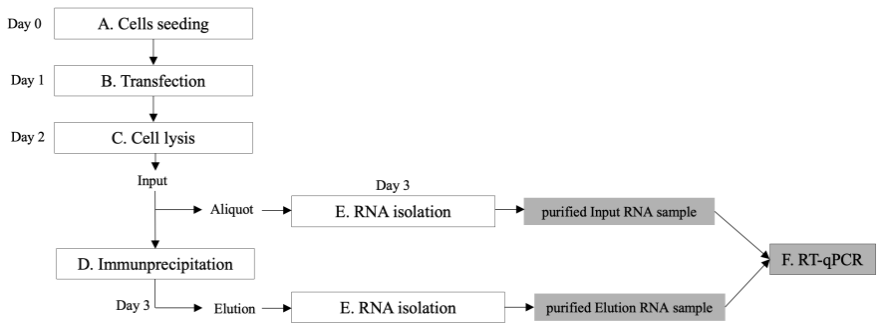
Overview of the protocol steps

The protocol here presented is designed for analysis of three conditions in a single experiment. If a different number of conditions is required, adapt volumes accordingly. A flowchart of the procedures is shown in Figure 1. See General note 1.

- **Seed 1 × 10^6^ cells onto a 15 cm dish per condition such that cells are confluent after 24 h**
- **Electroporation**
*Note: Carry out all procedures under a laminar flow hood and follow standard tissue culture procedures.*
  1. Prepare materials for the electroporation.
    - Place medium, trypsin, and HBSS solutions at 37 °C.
    - Place the electroporation tube into the holder in the Neon Electroporation machine.
    - Pipette 3 mL of E2 electrolytic buffer from Neon transfection system kit into the electroporation tube.
    - Prepare 3 × 1.5 mL tubes containing 1 mL of cell medium and keep at 37 °C. These are required later to enable recovery of cells following electroporation.
    - Prepare 3 × 1.5 mL tubes each containing 3–5 μg of plasmid DNA.
    - Prepare 3 × 1.5 mL tubes containing 1 mL of PBS. These are required later to rinse the electroporation pipette tip before moving to the next electroporation condition.
    - Select the transfection program: voltage 1,150 V, width 30 ms, 2 pulses.
    - Attach a gold electroporation tip to the electroporation pipette.
  2. Detach cells from 15 cm plates.
    - Remove medium and wash cells in 10 mL of PBS.
    - Remove PBS and add 1.5 μL of warm trypsin.
    - Incubate at 37 °C for 3–5 min and then add 15 mL of warm medium.
  3. Transfer cells to a 50 mL Falcon tube.
  4. Centrifuge at 200× *g* for 3 min at room temperature.
  5. Wash cells in 10 mL of PBS and centrifuge again at 200× *g* for 3 min.
  6. Remove PBS and wash again in 1 mL of PBS, transferring cells to 1.5 mL tubes.
  7. Centrifuge using MiniSpin at 300× *g* for 1 min at room temperature.
  8. Remove PBS and equilibrate cells in HBSS by resuspending cell pellet in 1 mL of warm HBSS.
  9. Centrifuge using MiniSpin at 300× *g* for 1 min.
  10. Resuspend cells in 330 μL of warm HBSS and transfer into appropriate tube with plasmid DNA.
*Note: Electroporation is performed in three rounds per condition. For this, use 100 μL per electroporation round plus excess to avoid formation of bubbles during pipetting.*
    **Caution:** Air bubbles could cause arcing during electroporation.
  11. Use the electroporation pipette to transfer cells to the Neon electroporation tube. Perform electroporation. Transfer electroporated cells to 1.5 mL tube with 1 mL of warm medium. Gently invert tube and place at 37 °C. **Caution:** When pipetting electroporated cells into tube with warm medium, avoid contact between the pipette tip and the medium as this could cause arcing due to presence of FBS in the medium.
  12. Repeat step B11 two more times for the same condition.
  13. Before moving to the next condition, rinse the electroporation pipette tip in PBS multiple times.
  14. Centrifuge electroporated cells using MiniSpin at 300× *g* for 1 min.
  15. Resuspend in 1 mL of warm medium, plate in 10 cm dishes (one dish per condition) with final 10 mL of medium and incubate at 37 °C for 24 h.
  16. Clean and dispose of electroporation kit components according to the manufacturer’s instructions. **Critical:** At sections C–F, strictly follow standard procedures for handling RNA. Treat work surfaces and pipettes with 70% ethanol and RNaseZap. Carry out all procedures on ice and always keep samples and solutions on ice. See General note 2.
- **Cell lysis**
  1. Prepare solutions.
    - 35 mL of PBS + CHX 0.1 mg/mL
    - 2.5 mL of lysis buffer
    - 1.2 mL of TBS 1×
  2. Add CHX at a final concentration of 0.1 mg/mL to electroporated cells and incubate at 37 °C for 15 min.
  3. Place plate on ice and wash cells once quickly with 10 mL of ice-cold PBS + CHX 0.1 mg/mL.
  4. Scrape quickly in 1 mL of ice-cold PBS + CHX 0.1 mg/mL while keeping plate on ice.
  5. Transfer cells into 1.5 mL tubes and centrifuge using MiniSpin at 300× *g* for 5 min at 4 °C.
  6. Resuspend cell pellets in 200 μL of ice-cold lysis buffer and incubate on ice for 15 min.
  7. Centrifuge using MiniSpin at 12,000× *g* for 15 min at 4 °C.
  8. Transfer 180 μL of supernatants (Input) into new 1.5 mL tubes.
*Note: Transfer less than 200 μL of supernatant to avoid collection of pelleted material.*
  9. Make a 10 μL aliquot for RNA isolation. Keep at 4 °C and perform RNA isolation (see section E) at the same time as the eluted RNA (see section D, steps 9–10).
- **Immunoprecipitation**
*Note: Use 40 μL of beads slurry per condition. Beads need to be mixed to achieve a homogenous suspension. Handle beads according to the manufacturer’s instructions. Use a magnetic rack to separate beads from solutions.*
  1. Transfer 120 μL of beads slurry to a 1.5 mL tube. Remove solution in which beads were stored.
  2. Wash beads twice in 600 μL of ice-cold TBS 1×.
*Note: Gently invert tube 4–5 times to wash beads.*
  3. Add 750 μL of ice-cold lysis buffer and split equally into 3 × 1.5 mL tubes.
  4. Add 170 μL of Input samples to respective tube containing beads.
  5. Incubate tubes at 4 °C overnight gently rotating at 10–30 rpm.
  6. Prepare solutions.
    - 3 mL of wash buffer.
    - 600 μL of puromycin elution buffer or 600 μL of 3× Flag peptide elution buffer.
  7. Discard supernatants (flowthrough).
  8. Wash beads twice with 0.5 mL of ice-cold wash buffer.
*Note: Gently invert tube 4–5 times to wash beads.*
  9. Perform elution. See General note 3.
    - Elution by puromycin: add 200 μL of puromycin elution buffer to each tube containing beads and incubate at 4 °C for 30 min gently rotating at 10–30 rpm. Collect Elution samples and keep on ice for RNA isolation to be performed straight after.
      or
    - Elution by 3× Flag peptide: add 100 μL of 3× Flag peptide elution buffer to each tube containing beads and incubate at 4 °C for 30 min gently rotating at 10–30 rpm. Collect eluates and repeat step. Collect eluates again and pool with previous respective eluates (total volume of elution samples 200 μL). Keep on ice for RNA isolation to be performed straight after.
  10. Perform RNA purification as in section E.
- **RNA isolation**
  1. Isolate RNA using Monarch RNA Cleanup kit according to the manufacturer’s instructions. These include a step of DNA digestion. Elute in 30 μL of ice-cold nuclease-free H_2_O.
  2. Assess RNA concentration and purity using the NanoDrop.
  3. Add EDTA at final concentration between 0.1 and 1 mM to samples.
*Note: This is optional. However, addition of EDTA may reduce the activity of any contaminating RNases. Make sure that EDTA is compatible with both the RNA isolation method and the reagents for downstream RT-qPCR.*
    **Pause Point:** Purified Input and Elution samples can be stored at -80 °C long term.
- **One-step RT-qPCR**
  See General note 4.
  1. Thaw RT-qPCR kit components, primers, and purified RNA samples on ice.
  2. Prepare a reaction master mix according to the manufacturer’s instructions. For three conditions, prepare the equivalent of 20 reactions (includes one extra reaction to account for pipetting errors).
*Note: Quickly vortex and spin all reaction reagents before use.*
  3. Distribute master mix to 19 wells of a 96-well plate.
  4. Distribute each RNA sample to three wells.
  5. Add a volume of H_2_O equivalent to the volume of RNA samples in a single well to the well corresponding to the no-template control reaction. **Critical:** Avoid formation of bubbles while pipetting during steps F3–F4. This might interfere with the amplification reaction.
  6. Cover plate with optical adhesive film.
  7. Quickly spin the plate to collect drops at the bottom of wells and to remove potential bubbles.
  8. Cover plate with darkening foil while setting up RT-qPCR machine.
  9. Load plate onto the RT-qPCR machine and start reaction. Perform one-step RT-qPCR according to the manufacturer’s instructions.

## Data analysis

Translation efficiency (TE) is calculated using the percent input method, which compares the amount of target mRNA measured in the Elution fraction to the total amount of the target mRNA in the Input fraction. For this, first the ∆Cq is calculated as follows:

∆Cq = Cq(Elution) - [Cq(Input) - Log_2_(DF)]

where DF is dilution factor (e.g., if 5% of total Input is used for RT-qPCR, the DF is 20). TE of reporter mRNA is then calculated as:

% Input = 100 × 2^-∆Cq^

**Figure 2.**
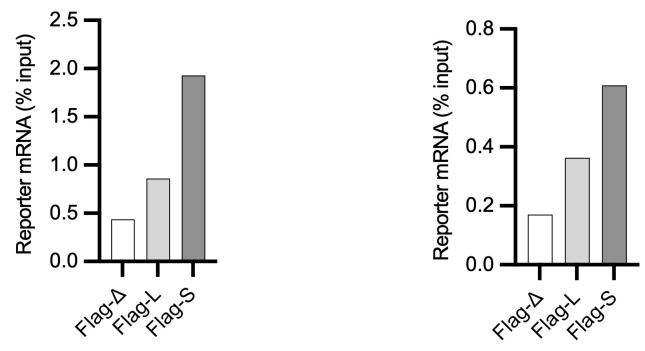
Quantification by RT-qPCR of indicated reporter mRNAs in an experiment designed to test the role of different 3′ untranslated regions (UTR) configurations (D, L, S) of Aurora kinase A (AURKA), eluted via puromycin (left) or 3× Flag peptide (right). Results representative of three biological replicates. See Cacioppo et al., 2023 for details of the experiment.

Representative data can be found in Figure 2. For each experiment, three technical replicates and three biological replicates should be performed. Appropriate controls for the experiment should be used, like using an untagged reporter protein (see Cacioppo et al., 2023) or untransfected cells.

**Representative data**

## Validation of protocol

This protocol was validated in Cacioppo et al. (2023). eLife. Differential translation of mRNA isoforms underlies oncogenic activation of cell cycle kinase Aurora A.

## General notes and troubleshooting

**General notes**

1. Other standard procedures for transfection (e.g., lipofectamine transfection) and cell lysis can be used. Number of cells to transfect per condition should be adapted to transfection efficiency or to the estimated expression levels of the reporter protein and determined empirically for the selected cell type. The cultures should be sub-confluent on the day of harvesting to avoid contact inhibition of translation. Furthermore, in some cell types and under particular states (e.g., genotoxic or proteotoxic stress), ribosomes may exist in stalled forms, suggesting that association of mRNA with ribosomes may not be indicative of active translation in these cases.
2. Make sure to optimise conditions. As for any procedure that involves handling and quantifying RNA, empirical tests may be required to find optimal conditions. Alternative methods of RNA isolation and RT-qPCR kits could be used, but conditions must be optimised accordingly.
3. To read about the action of puromycin, consult Blobel and Sabatini (1971) and Aviner (2020). It is important to note that, although being cost efficient and potentially eluting cleaner RNA, puromycin-induced release of ribosomes may be more variable upon conditions as salt, temperature, ribosome state, presence of CHX, or nucleic or amino acid sequence motifs, than 3× Flag-mediated elution. For this, it is recommended to initially test both elution methods in parallel, in order to find the more suitable for one’s own experimental aims and conditions.
4. It is highly recommended to perform RT-qPCR as soon as possible, to avoid alterations in the composition of the RNA extracts due to eventual RNA degradation. The Minimum Information for Publication of Quantitative Real-Time PCR Experiments (MIQE) guidelines (Bustin et al., 2009) should be followed when performing RT-qPCR experiments. One-step RT-qPCR can be replaced by sequential cDNA synthesis and RT-qPCR.
5. To control for efficiency of IP and for optimization purposes, RT-qPCR could be performed with primers to housekeeping genes (e.g., *ACTB, GAPDH*). In addition, RNA can be isolated from the Flowthrough, as well, and analysed in parallel with Input and Elution samples.
6. The assay could be adapted to immunoprecipitate nascent chains using other tags different from Flag tag. Use of small peptide tags is recommended to minimise possible influences on translation efficiency.
7. It is important to note that cell lysates typically contain a higher ratio of mature to nascent reporter protein. Although we have no evidence of this, it may be possible that the mature reporter protein interacts with ribosomes or mRNA outside the context of active translation (e.g., in RNA granules), and this could interfere with the interpretation of results. Furthermore, reporter expression levels will depend on transfection yield, overall translation rates, and time from transfection to harvesting. Therefore, the conditions should be determined empirically. A small-scale transfection experiment could be performed, harvesting samples at multiple time points, and measuring expression by anti-Flag immunoblotting.

**Troubleshooting (see Table 1)**

**Table 1.** Troubleshooting

Issue	Probable cause	Possible Solution
Low RNA yield	RNase contamination	• Ensure all instruments, workspaces, and reagents are RNase-free. • Change gloves routinely. • Use filtered pipette tips. • Pre-chill all tubes and buffers before use. • Increase volume of RNase inhibitor. • Perform procedures under a laminar flow hood.
Low IP efficiency (see General note 5)	Low electroporation efficiency	• Make sure to warm medium and solutions at 37 °C. • Make sure cells are not stressed, damaged, or contaminated by *Mycoplasma.* Cell density should not be too low or too high and fresh cultures should be used. • Make sure plasmids have high degree of purity and that a volume < 1/10 of volume of the cell suspension is used. • Electroporation conditions (voltage, width, pulses) might need to be optimised, especially if using a different cell line.
	Immunoprecipitated target RNA is too low	• Combine Elution RNA samples from multiple technical replicates. • Increase amount of beads slurry. • Increase stringency of washes. • Increase duration of the elution step.
	Low purity of Input/Eluted RNA	• Increase volume of RNase inhibitor. • Make sure samples are not contaminated by genomic DNA.
	Non-specific binding to beads	• Increase stringency of washes. • Decrease volume of lysate used. • Coat beads with bovine serum albumin prior to IP.
	Issues with RT-qPCR	• Make sure primers are designed to be optimal. Primer pairs should have amplification efficiency of 90%–110%. • Make sure pipetting is consistent and accurate. • Follow RT-qPCR kit manufacturer’s instructions on maximum amount of template RNA to use per reaction. • General RT-qPCR troubleshooting applies.
